# Cognitive and Social Rehabilitation in Schizophrenia—From Neurophysiology to Neuromodulation. Pilot Study

**DOI:** 10.3390/ijerph17114034

**Published:** 2020-06-05

**Authors:** Renata Markiewicz, Beata Dobrowolska

**Affiliations:** 1 Psychiatric Nursing Department, Faculty of Health Sciences, Medical University of Lublin, 20-081 Lublin, Poland; renata.markiewicz@umlub.pl; 2 Chair of Development in Nursing, Medical University of Lublin, Poland Faculty of Health Sciences, 20-081 Lublin, Poland

**Keywords:** schizophrenia, serum neurotrophic factor (BDNF), event-related potentials (ERP), QEEG Biofeedback

## Abstract

The aim of this pilot study was to analyse the influence of Galvanic Skin Response (GSR) Biofeedback training in a group of 18 men with schizophrenia at the remission stage. The results were verified according to: Positive and Negative Syndrome Scale (PANSS), Acceptance of Illness Scale (AIS), Self-efficacy Scale (GSES), Beck Cognitive Insight Scale (BCIS) scales, Colour Trial Test (CTT-1, CTT-2), d2 psychological tests, Quantitative Electroencephalogram (QEEG) Biofeedback, auditory event-related potentials (ERPs), and serum levels of brain-derived neurotrophic factor (BDNF). The results were compared in the same patients after 3 months. Statistically significant changes were noted in results for the variables on the PANSS scale. For the BDNF variable, a statistically significant increase occurred, indicating that GSR Biofeedback training may influence serum levels of the neurotrophic factor. Statistically significant changes were noted in results for the variables on the BCIS, AIS, and GSES indicating an improvement in the cognitive and social functioning. Changes were noted for results for theta/beta and theta/Sensory Motor Rhythm (SMR) ratios, which indicate an improvement in concentration and attention. Changes were noted for the N1 wave amplitude in the frontal brain region (F-z), and for the P2 wave latency in the central brain region (C-z), which indicates an improvement in the initial perceptual analysis. The use of GSR Biofeedback in a group of patients with schizophrenia gives interesting results, but requires further in-depth research.

## 1. Introduction

Schizophrenia is a disorder with a negative effect on the social functioning of affected patients. It is characterised by multifactor pathogenesis and, frequently, a recurrent course [1]. Productive symptoms predominate during the period of disorder exacerbation, while deficit symptoms prevail during its remission. Both positive and negative symptoms result from the disrupted activity of different areas of the brain [2]. Published reports indicate an important role of the frontal and temporal regions, limbic and medial structures, and of the basal ganglia [1,3,4]. Dysfunctions in the prefrontal region are associated with working memory, concentration, emotions, and executive functions [1,5,6], which influence patients’ functioning and quality of life [1,5,7,8,9,10].

Schizophrenia, as a disorder with a varying course, requires multidirectional therapeutic actions. Undoubtedly, regular pharmacological therapy that eliminates psychotic symptoms remains the primary form of treatment [11,12,13]. However, the supplementary form, which improves cognitive functions, is equally important.

Ever more interesting methods are sought amongst different forms of rehabilitation to enable the patient to be actively involved in the process of improving these functions in a simple way. One of such methods is Galvanic Skin Response (GSR) Biofeedback (GSR-BF), which uses feedback between the patient’s mental condition and the neurophysiological function for the deliberate control of mental and physiological functions on the basis of electrodermal activity of the skin.

Existing research suggests that the majority of schizophrenia patients have problems with concentration, attention, self-control, emotions, and social communication [14,15,16,17,18]. A diagnosis of these deficits forms a foundation for the therapy plan and duration, and for the selection of exercises [19]. Only correctly planned therapy guarantees a neuronal reorganisation and changes in synaptic connections (priming process). Kossut is of the opinion that the effect of changes can only be achieved when it is induced by a specific stimulus [20].

Among various forms of neurotherapy, such as trans-cranial magnetic stimulation (TMS), transcranial direct-current stimulation (tDCS), or electroconvulsive therapy (ECT), Biofeedback (BF) is gaining in popularity as a non-invasive method, which allows for the patient to voluntarily control their mental and physiological functions by using feedback between their mental condition and neurophysiological activity. Increasing reports indicate that BF therapy produces positive outcomes in patients with anxiety disorders, depression disorders, suicidal tendencies, bipolar disorder, and schizophrenia [21,22,23,24,25].

Various forms of BF are distinguished, depending on the type of brain–computer interface (BCI) used: GSR-BF (skin-galvanic reaction), EEG-BF (brain activity), EMG-BF (muscle response), or HRV-BF (heart rate). In the present pilot study, we assessed the outcomes of GSR-BF training in schizophrenic patients. The aim of the study was to determine whether GSR-BF training leads to neurophysiological changes in the subjects and whether these changes affect their cognitive and social functioning.

## 2. Materials and Methods

The convenience sample of 18 men with schizophrenia enrolled in the study underwent therapy that was based on the GSR-BF. The group was examined twice, at a baseline (Exam. 1) and after three months of therapy (Exam. 2). It was assumed that there were differences in the cognitive and social functioning of schizophrenia patients before and after training, which would be shown by the research methods and tools used. The study hypothesis assumed that GSR-BF training would prove to be a regulative control over neurophysiological mechanisms, and the obtained parameters would show an improvement in subjects’ cognitive and social functioning.

The study used:

1. diagnostic CTT test to analyse the frontal dysfunction, the CTT-1 version determined the visual performance and psychomotor speed (alternate joining of coloured numbers in a string from 1–25), the CTT-2 version determined the performance skills and working memory (alternate joining of numbers with a simultaneous selection of a colour sequence in a string from 1 to 25) [26];
2. d2 test of attention analysing the speed (amount of material processed in the specific time), quality (work precision and errors made) and persistence indicating features of behaviour during work (irritation, stability of work or lack of it, discouragement, fatigue); the level of concentration was a result of interaction of these behaviours, and a product of the stimulus and control coordination [27];
3. PANSS, a scale evaluating psychopathological symptoms of schizophrenia [28];
4. Beck Cognitive Insight Scale (BCIS) [29];
5. Acceptance of illness scale (AIS) of Felton, Revenson and Hinrichsen [30];
6. Self-efficacy scale (GSES) of Schwarzer and Jerusalem [30]; and,
7. QEEG-BF in terms of amplitudes and frequency ratios [31].

The laboratory parameter of neurotrophic factor BDNF was determined following blood sampling into a clot tube while using a non-contact method. The factor serum levels were determined with the immunoenzymatic technique ELISA (Human BDNF ELISA kit, R&D Systems, Minneapolis, MN, USA). The neuropsychological evaluation was performed by a psychologist, and the BDNF levels were determined by a laboratory diagnostician.

The tools used in the experiment were to prove therapy effects in:

- reduction in cognitive deficits (CTT-1, CTT-2, and d2 tests, neurotrophic factor, BDNF);
- reduction in positive (P) and negative (N) symptoms (PANSS scale);
- improvement in social adaptation (AIS, GSES, and BCIS scales);
- change in brain activity (QEEG Biofeedback);
- change in levels of the serum neurotrophic factor (BDNF); and,
- change in auditory event-related potentials (ERP).

### 2.1. Criteria for Patient Inclusion in the Study

The patients were recruited from among the day ward patients. They received information regarding the therapy and the process of collecting data for the study. Patients who agreed and met the inclusion criteria took part in the study after setting a training schedule.

The inclusion criteria were: patient’s consent, male gender, clinical diagnosis of schizophrenia (DSM V), patient’s age within a range of 18–50 years, dextrality, no neurological diseases (active and in the past), and excluded mental disability, dementia, or alcohol addiction.

Before entering the experiment, all men were in remission, i.e., remained in a stable mental state without psychotic (productive) symptoms, and the remission period in the subjects lasted 1.5 years. All of the subjects took atypical neuroleptics, both before entering the study and during treatment. A sudden change in treatment and a worsening of mental state were the reason for exclusion from the study. Trainings were carried out twice a week. Prior to therapy, the level of cognitive deficits (thinking, memory, concentration) was assessed based on the CTT and d2 tests. Both tests provided information regarding the level of occurring disorders based on the tasks performed.

Men were included in the study in order to eliminate gender differences (mainly hormonal differences) [32].

### 2.2. Apparatus

The GSR-BF training sessions were conducted in the following modules: CENTER (relaxation), BALANCE (concentration), and INSECTS (self-control), while using the Digi-Track apparatus (Elmiko-Medical Company, Warsaw, Poland). The training sessions were conducted twice a week for three months. Each subject obtained 24 measurements in three modules (72 measurements in total). The tests were performed in accordance with the approved schedule; the training was conducted in a sound-proof room, at a specified time, after a morning meal. For one hour before the test, the patients did not drink coffee or smoke. The measurements were conducted using the exosomatic method with DC (direct current) using electrodes that were inserted on index and ring fingers of the left hand and connected to the device presenting successive training modules. The training in individual modules was presented on the monitor screen, and the patient performed exercises in accordance with the instruction (no protocol applied).

The task of the respondents performing the exercise on the CENTER module was to achieve relaxation (through breath, heart rate), which was reflected in the graphic image on the monitor screen (the circle was filled with numerous bubbles), the greater the relaxation, the faster the patient performed the task and went to the next level of the module. Training on the BALANCE module concerned tasks that were related to improving concentration. The task of the respondents was to obtain the state of maximum concentration, as evidenced by the placing and holding a ball in the middle of a tilting board. The task on the INSECT module was to obtain a state of internal balance between cognitive and executive functions. The subjects’ task was to recognize moving and hidden insects on the monitor screen and clicking on them with the mouse. The slow movement of insects demonstrated the gradual achievement of internal balance (relaxation vs. activation) during training, which facilitated task realisation.

The GSR apparatus (Elmiko-Medical Company, Warsaw, Poland) registered neurophysiological changes, which determined the psychophysical condition of the subjects on the basis of their skin resistance. The training time was determined by a computer program, for the CENTER and BALANCE modules it was 5 min, for the INSECT module 10 min. Each session ended with the graphic recording of the results being achieved by the patient. 1296 measurements were obtained in total, 72 for each subject.

The potentials were tested using the Cognitrace apparatus. 21 cup electrodes (international 10–20 Electroencephalogram system with ear electrodes (ground and reference)) —Fpz, Fz, Cz, Pz, Oz, Fp1, Fp2, F3, F4, C3, C4, P3, P4, O1, O2, F7, F8, T3, T4, T5, T6, two ear electrodes A1 and A2, and GND, were attached to the patient’s head. The patient stayed in a separate, dark room. The test was performed with a subject in a sitting position, with eyes closed, and wearing earphones through which the acoustic stimuli were delivered in accordance with the oddball paradigm regimen (a series of tones of different frequency (1000 Hz and 2000 Hz) of ca. 70 dB for ca. 100 ms, in a random sequence). The P300 test, determining exogenous cognitive potential, was performed twice. One test lasted 3 min. and 20 s and contained 80% of frequent stimuli and 20% of rare (important) stimuli marked by the patient by pressing a button. The measurements in the studied group were performed twice.

A quantitative Electroencephalogram (QEEG) was performed in each patient, three months apart, before and after therapy. QEEG BF, concerning amplitudes and frequencies of individual waves, was performed while using the Digi-Track apparatus from (Elmiko-Medical Company, Warsaw, Poland). The patients had two electrodes installed, in the Fz and Cz regions, which registered the brain function in these regions, and the Fast Fourier Transform (FFT) algorithm transformed the raw EEG recording into frequencies for statistical processing (a so-called QEEG power spectrum). In the studied group, the brain rhythm from two selected areas was evaluated twice [20,31,33,34,35].

### 2.3. Statistical Analyses

The measurement results obtained were statistically analysed. The values of the analysed measurables were presented as a mean value and a standard deviation. The sociological and demographic parameters were presented as numbers and percentages. The results before (Exam. 1) and after (Exam. 2) therapy were compared with the Student’s t-test for dependent samples. A confidence level of *p* < 0.05 was assumed as indicating statistically significant differences. The coefficient of variation (CV%) was also calculated. The database was developed, and the statistical tests were conducted while using the Statistica 9.1 (StatSoft, Krakow, Poland) software.

### 2.4. Ethical Issues

The study protocol was accepted by Bioethics Committee—approval no. KE-0254/35/2016. All the patients invited to the study gave written consent after being informed about the study aims and protocol.

### 2.5. Patients Characteristics

The mean age in the studied group of men was 37 years (SD = 6.38), with education at the elementary (four patients), vocational (five patients), and secondary (nine patients) level. Fourteen people lived in large cities (over 100 thousand inhabitants), two people lived in smaller towns (below 100 thousand inhabitants), and two people lived in rural areas. Thirteen men included in the study lived together with their parents and five patients lived alone. Only one man in the group was married, 15 patients were single, and two were divorced. Sixteen men declared that they had no children, and two stated that they had one child. Almost all of the subjects did not work, 13 of them received a disability allowance, and five received the unemployment benefit. Only one subject worked in a job consistent with his education. No history of mental illness was reported on the mother’s side by 18 men, and on the father’s side by 17 men. Such history on the mother’s side was indicated by only one patient, and on the father’s side by two subjects. Two-thirds of the subjects stated that they underwent regular treatment, while one-third reported irregular treatment. The most common cause of disease recurrence was occasional use of alcohol, which caused a lack of regularity in taking medicines (five patients) and discontinuation of medicines (four people). Half of the men included in the study said that there was no clear reason for disease recurrence. In the men included in the study, the mean number of hospitalisations was seven stays (SD = 4.8). Suicide attempts were negated by 12 subjects and disclosed by seven subjects.

Half of the patients stated that they were treated for concurrent diseases, with the most common being cardiovascular diseases and skin diseases. The remaining patients did not declare any other diseases. All of the subjects took atypical neuroleptics and were right-handed. Only 1/3 of the subjects were addicted to nicotine (10 cigarettes a day).

## 3. Results

A comparative analysis of the measurements obtained was conducted at two time points, at the beginning of the therapeutic experiment (Exam. 1) and after (Exam. 2) the training, in order to verify the assumptions made in the study and determine the influence of GSR-BF therapy in the patients diagnosed with schizophrenia. The measurements obtained are presented in Table 1, containing only statistically significant differences.

In the examinations, no statistically significant changes were noted in the measurement results for psychological tests CTT-1, CTT-2, and d2, which is why these data are not shown in Table 1.

Statistically significant changes were noted in the measurement results for the variables on the PANSS scale. On all analysed scales: POS (M = 9.06; SD = 2.04 vs. M = 7.50; SD = 2.23), NEG (M = 13.94; SD = 3.92 vs. M = 11.83; SD = 4.48), GEN (M = 24.83; SD = 3.35 vs. M = 22.61; SD = 3.71), and TOT (M = 47.83; SD = 8.49 vs. M = 41.94; SD = 9.64), a statistically significant drop occurred in the results obtained.

For the BDNF variable, a statistically significant increase in the measurement occurred (M = 44.78; SD = 10.69 vs. M = 55.50; SD = 10.76).

For variables concerning the scales used, statistically significant changes were noted in measurement results for the variables on the BCIS (reflectiveness) (M = 22.72; SD=4.80 vs. M = 25.72; SD = 3.14), AIS (illness acceptance) (M = 22.67; SD= 8.95 vs. M = 26.44; SD = 6.46), and GSES (self-efficacy) (M = 23.78 SD = 5.43 vs. M = 27.61; SD = 5.09). On these scales, a statistically significant increase in the results obtained occurred.

Changes were also noted for the measurement results for theta/beta (M = 1.92; SD = 0.57 vs. M = 2.29; SD = 0.88), and theta/SMR (M = 2.07; SD = 0.64 vs. M = 2.37; SD = 0.8) ratios.

For variables concerning event-related potentials, statistically significant changes were noted for the N1 wave amplitude (M = −3.95; SD = 2.53 vs. M = −5.36; SD = 1.93) in the Fz region, and statistically significant changes (shortening) were noted for the P2 wave latency (M = 209; SD = 14.81 vs. M = 196; SD = 18.27) in the Cz region.

## 4. Discussion

Our study is of a pilot nature and, as far as we know, there have been no studies using this therapy and research tools in patients with schizophrenia. Therefore, it is quite difficult to compare research results for this particular group of patients and this kind of Biofeedback. Therefore, the authors tried to compare the obtained results in our study for individual indicators with results from research of other authors.

The data obtained indicates that the therapy used resulted in a statistically significant change in the measurement results for the variables on the PANSS scale. For all analysed scales, a statistically significant drop occurred in the results obtained, which implied a decrease in the positive (e.g., excitement, suspicion, hostility) and negative symptoms (e.g., emotional and social withdrawal, difficulties in abstract thinking) due to therapy. The results were confirmed in studies that were conducted by Schell, whose research implies that a high level of stimulation and excessive activity are positively correlated with the cognitive processing, an increase in conductivity, and a galvanic response [25]. Other researchers also confirmed the results that were obtained by Schell and support the relationship between an increase in the GSR signal and stimulation [36,37,38]. The results obtained in this study are consistent with the results obtained by those authors.

For the BDNF variable, a statistically significant increase occurred for the measurement of neurotrophic factor serum levels, which is possibly related to a positive influence of GSR-BF therapy. Additionally, although the reports on this subject are scarce, it should be assumed that the observed effect might be a result of the therapy conducted. This is confirmed by the results obtained by Ghaziri, who analysed changes occurring in the white and grey matter during the neurofeedback training (NFT) in healthy subjects using f-MRI. The author demonstrated that the maintenance of attention during the training is associated with structural changes in pathways in the white matter, which is responsible for cognitive capacities [39]. Vinogradov also confirmed this, who indicates a relationship between cognitive training and an increase in the blood serum level of BDNF [40]. It is probable that the effect of stimulus consistency and the rise in the neurotrophic BDNF factor level result from the increase in the interplanar transfer of information [41].

Mattson and Mennerick are of the opinion that the lack of structured rehabilitation intensifies negative symptoms and causes a decrease in the neurotrophic factor BDNF levels, which, in turn, results in poorer cognitive and social functioning of patients diagnosed with schizophrenia. These authors state that the lack of energy transformations decreases biochemical processes, inhibits neurotransmitter production, and reduces BDNF synthesis [42,43]. Similar results were obtained by Heitz, who analysed the relationship between BDNF and the disease stage: prodromal, the first psychotic episode, and a chronic condition. The author demonstrated a relationship between the disease stage and BDNF level. He obtained the lowest BDNF values for chronically ill people, with decreased cognitive functions [44].

For variables concerning the scales used, statistically significant changes were noted in measurement results for variables on the BCIS A (reflectiveness), AIS (illness acceptance), and GSES (self-efficacy) scales. For all scales, a statistically significant increase in the results obtained occurred, and this might indicate an improvement in the cognitive and social functioning of the subjects. These results are consistent with those that were obtained by Ahmed et al. [45] and Vinogradov et al. [40], who are of the opinion that a positive correlation between the BDNF levels is associated with reasoning, problem solving, and general social adaptation of patients. Bowie et al. and Sartory et al. are of the opinion that schizophrenia patients react better to visual rather than audio training, and this may be a possible reason why they obtain better results for this type of therapy [46,47].

The analyses conducted so far show that systematic training affects the activity of specific brain areas by organizing them [48,49]. The modulation of brain waves, mainly beta1, alpha, and SMR waves, reduces cognitive deficits that are associated with memory, attention, and executive functions. The basic goal of BF therapy is to restore normal activity to dysfunctional brain areas. Many publications confirm this dependency, among others works by Trousselard et al. [50] and Scheinost et al. [51]. These authors believe that regulation of brain waves positively affects the levels of anxiety and uneasiness, symptoms that often accompany schizophrenia. Similar conclusions are made by Larsen [48], who claims that BF has special application in patients who show an adverse response to psychopharmacologic treatments and psychotherapy. It constitutes a therapeutic alternative with a positive prognosis for rehabilitation. Other researchers, Birbaumer et al. [16] and Mathiak et al. [52], compare BF self-regulation to the processes of learning and operant conditioning based on reinforcing and rewarding specific behaviours. They claim that these processes increase the involvement of the dopaminergic system, thus enhancing encoding in the reward pathway. Rota et al. reported interesting conclusions [53], who claim that BF-based training to activate the frontal region of the right inferior gyrus has an impact on speech modulation and processing. Positive effects of BF training have also been observed by Ruiz et al. [54], who report that BF training affects the perception of emotions in schizophrenia patients, and Naimijo et al., [55], who suggest that training has a desirable effect on executive functions.

In our current study, changes were also noted for the results of measurements of the theta/beta and theta/SMR ratios, which imply an improvement in concentration and attention. Although the number of available publications analysing the meaning of these ratios and their influence on the functioning of people diagnosed with schizophrenia is low, it might be assumed with some probability that certain data can suggest this effect. The studies by Gruzelier, who analysed attention, concentration, and memory in a group of artists on the basis of the alpha/theta, SMR/theta, and SMR/beta2 protocol, represent an example of such works. He demonstrated that actively conducted training improves cognitive processes in musicians and increases their artistic abilities [56,57]. He obtained similar results when examining healthy people on the basis of the SMR/beta 1 protocol. He proved a positive effect of therapy on self-control and reflective action (SMR), as well as concentration and decisiveness in problem solving (beta1) [58]. Egner analysed a group of healthy people undergoing Biofeedback EEG therapy on the basis of improvement in SMR, beta, and beta1 rhythms for attention processing. The author proved a strengthening effect of the therapy on the reaction speed, which was associated with an increase in the P3 wave amplitude [59]. Therefore, it appears probable that the use of specific Biofeedback protocols in schizophrenia patients may improve these processes.

In the future, QEEG might become a diagnostic marker, used for a diagnosis of brain activity on the basis of quantitative EEG analysis. Such an approach might enable obtaining information required for the rehabilitation process. Specialists in the field state that, because brain activity is described by a specific configuration of waves responsible for its functioning, their evaluation may facilitate defining the scope of neurotherapy [60,61]. Klimesh at al. [62] and Basar et al. [63] emphasize the great importance of delta, theta beta, and alpha waves in therapy of this type.

For variables concerning event-related potentials (ERP), a statistically significant change was noted for the N1 amplitude at Fz, implying an improvement in the stimulus identification, and statistically significant shortening of the P2 latency at Cz proving better analysis for the stimulus [64,65,66]. Although this study did not confirm a significant influence of GSR-BF training on a change in the P3 wave responsible for endogenous cognitive functions [67], it can be supposed that such a process could have been induced. Perhaps the time of the influences applied was too short to cause statistically significant changes. Similar results for the P2 wave were obtained by Kariofilis, who evaluated the influence of the cognitive training on auditory event-related potentials in schizophrenia patients. In her studies, she demonstrated a relationship between reduced P2 latency and neuropsychological indicators, as well as social and professional indicators [68].

It should be emphasized that our study has limitations. The limitations include the selection of sample, which was not random but rather based on convenience sampling methodology. Moreover, PANSS assessment was made by one of researchers, which could introduce selection bias. Another important limitation is the lack of a control group, which is planned in the next step of our project as this one is a pilot study. Additionally, the division of patients qualified for the study, depending on the cognitive deficits, would be an interesting solution when investigating the differences. However, it should be done on a much larger group of participants and taken into account, as in this pilot study: the level of education of the respondents, age, number of hospitalizations, and eliminate neurological diseases, mental retardation, dementia, alcohol, and drug addiction. In addition, psychological tests verifying the level of intelligence could be included. Indubitably, further investigations in a larger group of patients with control groups are needed to make consistent and firm conclusions. The present study is a pilot experiment, but, even at this stage, it makes a contribution, providing incentive for further work on this topic. From the point of view of the mentally ill, any method that improves their social functioning and quality of life is worth exploring in further detail.

## 5. Conclusions

Rehabilitation that is based on GSR-BF resulted with a regulative control over neurophysiological mechanisms, while the obtained parameters demonstrated an improvement in the cognitive and social functioning of the study subjects. The tools used in the experiment showed therapy effects in: reduction in positive and negative symptoms on the PANSS scale; increase in the brain derived neurotrophic factor (BDNF); increase in the theta/beta and the theta/SMR ratios in QEEG Biofeedback; and, improvement in cognitive and social functioning on AIS, GSES, and BCIS scales.

## Figures and Tables

**Table 1:** Comparative analysis of results obtained before therapy (Examination 1) and after therapy (Examination 2); latency (latency time in ms), and amplitude (mv, peak-to-peak).

Variable	Examination 1			Examination 2			Difference	Significance of Differences		Confidence Level	
	M	SD	CV%	M	SD	CV%		T	*p*	−95%	+95%
PANSS-POS	9.06	2.04	22.52	7.50	2.23	29.73	−1.56	10.719	<0.001	−1.86	−1.25
PANSS-NEG	13.94	3.92	28.12	11.83	4.48	37.87	−2.11	8.304	<0001	−2.65	−1.57
PANSS-GEN	24.83	3.35	13.49	22.61	3.71	16.41	−2.22	10.736	<0001	−2.66	−1.79
PANSS-TOT	47.83	8.49	17.75	41.94	9.64	22.99	−5.89	11.834	<0001	−6.94	−4.84
BDNF	44.78	10.69	23.87	55.50	10.76	19.39	10.72	−6.185	<0001	7.06	14.38
BCISS	22.72	4.80	21.13	25.72	3.14	12.21	3.00	−3.170	0.006	−5.00	−1.00
AIS	22.67	8.95	39.48	26.44	6.46	24.43	3.78	−2.547	0.021	0.65	6.91
GSES	23.78	5.43	22.83	27.61	5.09	18.44	3.83	−3.239	0.005	1.34	6.33
QEEG C-z theta/beta	1.92	0.57	29.69	2.29	0.88	38.43	0.37	−2.632	0.018	0.07	0.67
QEEG C-z theta/SMR	2.07	0.64	30.92	2.37	0.80	33.76	0.30	−2.358	0.031	0.03	0.57
F-z N1 (amplitude)	−3.95	2.53	64.05	−5.36	1.93	36.01	−1.41	2.588	0.020	−2.57	−0.26
C-z P2 (latency)	208.8	14.81	7.09	196.1	18.27	9.32	−12.77	2.643	0.018	−23.01	−2.52

M—mean value; SD—standard deviation; CV%—coefficient of variation; T—Student’s t-test; *p*—level of significance PANSS-POS—Positive and Negative Syndrome Scale—POSITIVE; PANSS-NEG—Positive and Negative Syndrome Scale—NEGATIVE; PANSS-GEN—Positive and Negative Syndrome Scale—GENERAL; PANSS-TOT—Positive and Negative Syndrome Scale—TOTAL; BDNF—brain derived neurotrophic factor; BCISS—Beck Cognitive Insight Scale; AIS—Acceptance of Illness Scale; GSES—Self-efficacy Scale; QEEG C-z theta/beta—attention factor of the central area; QEEG C-z theta/SMR—concentration factor of the central area; F-z N1 (amplitude)—amplitude of the first negative component of the central area; C-z P2 (latency—delay of the second positive component of the central area.

## Abbreviations

GSR, galvanic skin response; f MRI, functional magnetic resonance; quantitative changes in the EEG (theta/beta ratio, theta/SMR ratio); BDNF, non-glycosylated protein, brain derived neurotrophic factor; AIS, Acceptance of illness scale; GSES, Self-efficacy scale; BCIS, Beck Cognitive Insight Scale; CTT-1, test to analyse the frontal dysfunction, the CTT-1 version determined the visual performance and psychomotor speed; CTT-2, test to analyse the frontal dysfunction, the CTT-2 version determined the performance skills and working memory; d 2 psychological test, test of attention; ERP, event-related potentials; N1, first negative-going component; P2, second positive-going component; QEEG, quantitative EEG; PANSS, Positive and Negative Syndrome Scale; F-z, frontal brain region; C-z, central brain region; CNS, central nervous system; SCR_s_, skin conductance responses.

## References

[1] Kahn R. (2014). Why Kraepelin was right: Schizophrenia as a cognitive disorder. Neuropsychiatria i Neuropsychologia.

[2] Thomas A. (2005). Diagnostic categories or dimensions? A question for the diagnostic and statistical manual of mental disorders—Fifth Edition. J. Abnorm. Psychol..

[3] Borkowska A. (2012). Zaburzenia Funkcji Poznawczych w Schizofrenii: Aspekty Neuropsychiatryczne i Neuropsychologiczne [Cognitive Impairment in Schizophrenia: Neuropsychiatric and Neuropsychological Aspects].

[4] Heaton R., Gladsjo J., Palmer B., Kuck J., Marcotte T.D., Jeste D.V. (2001). Stability and course of neuropsychological deficits in schizophrenia. Arch. Gen. Psychiatry.

[5] Harvey P., Koren D., Rechenberg A., Bowie C. (2006). Negative symptoms and cognitive deficits what is the nature of their relationship?. Schizophr Bull..

[6] Hoff P., Beer D. (1992). Introductory remarks on the translation of Emil Kraepelins paper Die Erscheinungsformen des Irreseins (1920). Hist. Psychiatry.

[7] Bralet M., Navarre M., Eskenazi A., Lucas-Ross M., Falissard B. (2008). Interest of a new instrument to assess cognition in schizophrenia: The brief assessment of cognition in schizophrenia (BACS). Encephale.

[8] Mohamed S., Paulsen J., O’Leary D., Arndts S., Andreasen N. (1999). Generalized cognitive deficits in schizophrenia: A study of first-episode patients. Arch. Gen. Psychiatry.

[9] Riley E., McGovern D., Mockler D., Docu V.C., OCeallaigh S., Fannon D.G., Tennakoon L., Santamaria M., Soni W., Morris R.G. (2000). Neuropsychological functioning in first-episode psychosis evidence of specific deficits. Schizophr. Res..

[10] Saykin A., Shtasel D., Gur R., Kester D.B., Mozley L.H., Stafiniak P., Gur R.C. (1994). Neuropsychological deficits in neuroleptic naive patients with first- episode schizophrenia. Arch. Gen. Psychiatry.

[11] Schuepbach D., Keshavan M., Kmiec J., Sweeney J. (2002). Negative symptom resolution and improvements in specific cognitive deficits after acute treatment in first-episode schizophrenia. Schizophr. Res..

[12] Townsend L., Malla A., Norman R. (2001). Cognitive functioning in stabilized first episode psychosis patients. Psychiatry Res..

[13] Floresco S., Geyer M., Gold L.H., Grace A.A. (2005). Developing predictive animals models and establishing a preclinical trias network for assessing treatment effects on cognition in schizophrenia. Schizophr. Bull..

[14] Green M., Kern R., Braff D., Mintz J. (2006). Neurocognitive deficits and functional outcome in schizophrenia: Are we measuring the „right stuff”?. Schizophr. Bull..

[15] Brekke J., Raine S., Ansel M. (1997). Neuropsychological and psychophysiological correlates of psychosocial functioning in schizophrenia. Schizophr. Bull..

[16] Birbaumer N., Ruiz S., Sitaram R. (2013). Learned regulation of brain metabolism. Trends Cogn. Sci..

[17] Campo J., Merkelbach H., Nijman H., Yeates-Frederikx M., Allertz W. (2000). Skin conductance and schizophrenic symptomatology. Acta Neuropsychiatr..

[18] Iacono W., Lykken D., Peloquin L. (1983). Electrodermal activity in euthymic unipolar and bipolar affective disorders. Arch. Gen. Psychiatry.

[19] Braithwaite J., Watson D., Jones R., Rowe M. (2015). A Guide for Analysis Electrodermal. (EDA), Skin Conductance Responses (SCR_S_) for Psychological Experiments Activity.

[20] Kossut M. (1993). Mechanizmy Plastyczności Mózgu [Mechanisms of Brain Plasticity].

[21] Sarchiapone M., Gramaglia C., Iosue M., Carli V., Mandelli L., Serretti A., Marangon D., Zeppegno P. (2018). The association between electrodermal activity (EDA), depression and suicidal behaviour: A systematic review and narrative synthesis. BMC Psychiatry.

[22] Thorell L., Wolfersdorf M., Straub R., Steyer J., Hodgkinson S., Kaschka W. (2013). Electrodermal hyporeactivity as a trait marker for suicidal propensity in uni-and bipolar depression. J. Psychiatr. Res..

[23] Wolfersdorf M., Straub R., Barg T., Keller F. (1996). Depression and electrodermal response measures in habituation experiment. Results from over 400 depressed inpatients. Fortschr. Neurol. Psychiatr..

[24] Jandl M., Steyer J., Kaschka W. (2010). Suicide risk markers in major depressive dis order: A study of electrodermal activity and event-related potentials. J. Affect. Disor..

[25] Schell A., Dawson M., Rissling A., Ventura J., Subotnik K., Gitlin M. (2005). Electrodermal predictors of functional outcome and negative symptoms in schizophrenia. Psychophysiology.

[26] D’Elia L., Satz P., Uchiyama C., White T. (2012). Kolorowy Test Połączeń (Color Trials Test).

[27] Dajek E.R. (2012). Polska Standaryzacja Testu d2, Testu Badania Uwagi R. Brickenkampa (Polish Standardization of d2 Test, R. Brickenkamp’s Attention Test).

[28] Kay S., Fiszbein A., Opler L. (1987). The positive and negative syndrome scale (PANSS) for schizophrenia. Schizophr. Bull..

[29] Yu-Chen K., Yia-Ping L. (2010). The Beck cognitive insight scale (BCIS): Translation and validation of the Taiwanese version. BMC Psychiatry.

[30] Juczyński Z. (2012). Narzędzia Pomiaru w Promocji i Psychologii Zdrowia. (Measurement Tools in Health Promotion and Health Psychology).

[31] Thompson M., Thompson L. (2013). Neurofeedback. Wprowadzenie do Podstawowych Koncepcji Psychofizjologii Stosowanej [Neurofeedback. Introduction to the Basic Concepts of Applied Psychophysiology].

[32] Begliuomini S., Casarosa E., Pluchino N., Lenzi E., Centofanti M., Freschi L., Pieri M., Genazzani A.D., Luisi S., Genazzani A.R. (2007). Influence of endogenous and exogenous sex hormones on plasma brain-derived neurotrophic factor. Hum. Reprod..

[33] Rowan J., Tolunsky E. (2004). Podstawy EEG z Mini Atlasem [Basics of EEG with a Mini Atlas].

[34] Majkowski J. (1989). Elektroencefalografia Kliniczna (Clinical Electroencephalography).

[35] Smyk K. (2015). Teoria i Praktyka Terapii Neurofeedback. Materiały Szkoleniowe Ośrodka Kształcenia Medycznego AKSON (Theory and Practice of Neurofeedback Therapy. Training Materials of the AKSON Medical Training Center).

[36] Jayanthi A., Nivedha R., Vani C. (2015). Galvanic skin response measurement and analysis. IJAER.

[37] Zakeri S., Abbasi A., Goshvarpour A. The effect of creativity test on the galvanic skin response signal and detection with support vector machine. Proceedings of the International Conference on Research in Engineering Science and Technology.

[38] Noraziah A., Abdullah M.A.S., Aqtar N., Fakhreldin M.A.I., Wahab M.N.A. (2015). Greenvec game for skin conductivity level (SCL) Biofeedback performance stimulator using galvanic skin response (GSR) sensor. IJSECS.

[39] Ghaziri J., Tucholka A., Larue V., Blanchette-Sylvestre M., Reyburn G., Gilbert G., Lévesque J., Beauregard M. (2013). Neurofeedback training induces changes in white and gray matter. Clin. EEG Neurosci..

[40] Vinogradov S., Fisher M., Holland C., Shelly W., Wolkowith O., Mellon S. (2009). Is serum brain-derived neurotrophic factor a biomarker for cognitive enhancement in schizophrenia?. Biol. Psychiatry.

[41] Zvyagintsev M., Clemens B., Chechko N., Mathiak K., Sack A., Mathiak K. (2013). Brain networks underlying mental imagery of auditory and visual information. Eur. J. Neurosci..

[42] Mattson M., Mandsley S., Martin B. (2004). BDNF and 5-HT: A dynamic duo in age-related neuronal plasticity and neurodegenerative disorders. Trends Neurosci..

[43] Mennerick S., Zorumski C. (2000). Neural activity and survival in the developing nervous system. Mol. Neurobiol..

[44] Heitz U., Papmeyer M., Studerus E., Egloff L., Ittig S., Andreou C., Vogel T., Borgwardt S., Graf M., Eckert A. (2018). Plasma and serum brain-derived neurotrophic factor (BDNF) levels and their association with neurocognition in at-risk mental state, first episode psychosis and chronic schizophrenia patients. World J. Biol. Psychiatry.

[45] Ahmed A., Mantini A., Fridberg D., Buckley P. (2015). Brain-derived neorotrophic factor (BDNF) and neurocognitive deficits in people with schizophrenia: A meta-analysis. Psychiatry Res..

[46] Bowie C., McGurk S., Mausbach B., Patterson T., Harvey P. (2012). Combined cognitive remediation and functional skills training for schizophrenia: Effects on cognition, functional competence and real-world behavior. Am. J. Psychiatry.

[47] Sartory G., Zorn C., Groetzinget G., Windgassen K. (2005). Computerized cognitive remediation improves verbal learning and processing speed in schizophrenia. Schizophr. Res..

[48] Lawrine S.M., Whalley H.C., Abukmeil S.S., Kestelman J.M., Donnelly L., Miller P., Best J.J., Owens D.G., Johnstone E.C. (2001). Brain structure, genetic liability, and psychotic symptoms in subjects at high risk of developing schizophrenia. Biol. Psychiatry.

[49] Larsen S., Sherlin L. (2013). Neurofeedback: An emerging technology for treating central nervous system dysregulation. Psychiatr. Clin. North. Am..

[50] Trousselard M., Canini F., Claverie D., Cungi C., Putois B., Franck N. (2016). Cardiac coherence training to reduce anxiety in remitted schizophrenia, a pilot study. Appl. Psychophysiol. Biofeedback.

[51] Scheinost D., Stoica T., Saksa J., Papademetris X., Constable R.T., Pittenger C., Hampson M. (2013). Orbitofrontal cortex neurofeedback produces lasting changes in contamination anxiety and resting-state connectivity. Transl. Psychiatry.

[52] Mathiak K.A., Koush Y., Dyck M., Gaber T.J., Alawi E., Zepf F.D., Zvyagintsev M., Mathiak K. (2010). Social reinforcement can regulate localized brain activity. Eur. Arch. Psychiatry Clin. Neurosci..

[53] Rota G., Sitaram R., Veit R., Erb M., Weiskopf N., Dogil G., Birbaumer N. (2009). Self-regulation of regional cortical activity using real-time fMRI: The right inferior frontal gyrus and linguistic processing. Hum. Brain Mapp..

[54] Ruiz S., Lee S., Soekadar S.R., Caria A., Veit R., Kircher T., Birbaumer N., Sitaram R. (2013). Acquired self-control of insula cortex modulates emotion recognition and brain network connectivity in schizophrenia. Hum. Brain Mapp..

[55] Naimijoo P., Rezaei O., Feizzadeh Z. (2015). Neurofeedback training in schizophrenia: A study on executive functioning. Eur. Online J. Nat..

[56] Gruzelier J. (2009). A theory of alpha/theta neurofeedback, creative performance enhancement, long distance functional cennectivity and psychological integration. Cogn. Process..

[57] Gruzelier J., Hirst L., Holmes P., Leach J. (2014). Immediate effects of alpha/theta and sensory-motor-rythm feedback on music performance. Int. J. Psychophysiol..

[58] Gruzelier J. (2014). Differential effect on mood of 12-15 (SMR) and 15-18 (beta1) Hz neurofeedback. Int. J. Psychophysiol..

[59] Egner T., Gruzelier J. (2004). EEG biofeedback of low beta band components: Frequency-specyfic effects on variables off attention and event-related brain potentials. Clin. Neurophysiol..

[60] Wichniak A., Basińska-Starzycka A., Wierzbicka A., Jernajczyk W. (2016). Zastosowanie badań potencjałów wywołanych w ocenie funkcji poznawczych. (The use of event related potentials in the evaluation of cognitive functions). Wiad. Psychiatr..

[61] Ford J. (1999). Schizofrenia: The broken P300 and beyond. Psychophysiology.

[62] Klimesh W., Doppelmayr M., Schwaiger J., Winkler T., Gruber W. (2000). Theta oscillations and the ERP old/New effect: Independent phenomena?. Clin. Neurophysiol..

[63] Basar E., Barar-Eroglu C., Guntekin B., Yener G. (2013). Brain’s alpha, beta, gamma, delta and theta oscillations in neuropsychiatric diseases: Proposal for biomarker strategies. Suppl. Clin. Neurophysiol..

[64] Maurer K., Riederer P., Heinsen H., Beckmann H. (1989). Altered P300 topography due to functional and structural disturbances in the limbic system in dementia and psychoses and to pharmacological conditions. Psychiatry Res..

[65] Pfefferbaum A., Wenegrat B., Ford J., Roth W., Kopell B. (1984). Clinical application of the P3 component of event related potentials. Dementia, depression and schizophrenia. Electroenceph. Clin. Neurophysiol..

[66] Ferreira-Santos F., Silveira C., Almeida P., Palha A., Barbosa F., Margnes-Teixeira J. (2012). The auditory P200 is both increased and reduced in schizophrenia? A meta-analytic dissociation of the effect for standard and target stimuli in the oddball task. Clin. Neurophysiol..

[67] Szelenberger W. (2001). Potencjały Wywołane [Evoked Potentials].

[68] Kariofillis D., Sartory G. (2014). The effect of cognitive training on evoked potentials in schizophrenia. Schizophr. Res. Cogn..

